# Acquired Generalized Lipodystrophy as an Adverse Event of Combined Immune Checkpoint Inhibitor Therapy

**DOI:** 10.1210/jcemcr/luaf023

**Published:** 2025-02-10

**Authors:** Randol Kennedy, Calvin Rei L Macrohon, Mary Lourdes Grace V David, Meg Lee, April K S Salama, Afreen Shariff

**Affiliations:** Department of Endocrinology, Duke University Medical Center, Durham, NC 27710-1000, USA; Department of Endocrinology, Makati Medical Center, Makati City 1229, Philippines; Department of Endocrinology, Makati Medical Center, Makati City 1229, Philippines; Department of Endocrinology, Duke University Medical Center, Durham, NC 27710-1000, USA; Department of Endocrinology, Duke University Medical Center, Durham, NC 27710-1000, USA; Department of Endocrinology, Duke University Medical Center, Durham, NC 27710-1000, USA

**Keywords:** lipodystrophy, immune checkpoint inhibitor, immune-related adverse event

## Abstract

Acquired generalized lipodystrophy (AGL) is rarely associated with immune checkpoint inhibitors (ICIs). A few cases report associations with inhibitors to programmed cell death protein 1 (PD-1 inhibitors); however, association with combined immunotherapies has not been reported. We present a 48-year-old female with recurrent malignant melanoma who underwent 23 cycles of nivolumab, a PD-1 inhibitor, and 11 cycles of combined cytotoxic T lymphocyte associated antigen 4 (CTLA-4) inhibitor and PD-1 inhibitor ipilimumab-nivolumab. During the latter course of combination therapy, she experienced progressive weight loss and a dramatic change in body habitus over 3 to 6 months. Physical examination showed generalized loss of subcutaneous fat with protruding veins and muscular definition. Metabolic workup showed new-onset diabetes mellitus, a very low high-density lipoprotein, severe hypertriglyceridemia, and undetectable leptin/adiponectin levels. Whole-body fluorodeoxyglucose positron emission tomography performed for restaging and response assessment revealed generalized soft tissue edema and diffuse hepatic steatosis. An excisional skin biopsy identified changes consistent with involutional lipoatrophy/lipodystrophy. Treatment included insulin (average total daily dose 0.13-0.26 Units/kg/day) and a combination of lipid-lowering therapy (statins, fenofibrate, icosapent ethyl), which led to marked improvement in triglycerides and symptoms. This case further underscores the emerging challenges with endocrinopathies associated with checkpoint inhibitors.

## Introduction

Lipodystrophy syndromes are rare, congenital or acquired heterogenous disorders, characterized by partial or generalized deficiency and dysfunction of adipose tissue, resulting in a myriad of metabolic complications such as hyperinsulinemia, dyslipidemia, and metabolic dysfunction associated hepatosteatosis [1]. Due to the rarity of lipodystrophy syndromes, many clinicians are unfamiliar with the approach to diagnosis and management [1].

The use of immune checkpoint inhibitor (ICI) therapy has evolved the landscape of cancer therapy, since its introduction in 2011. With the expanding use of this class of immunotherapy, however, came the identification of many immune-related adverse events (irAEs) with endocrinopathies being among the most common [2].

Among the endocrinopathies, acquired generalized lipodystrophy (AGL) is an exceedingly rare consequence, with an incidence of only a few case reports. So far, these cases report a possible correlation with programmed cell death protein 1 (PD-1) inhibitor therapy. To our knowledge, this is the first reported case of AGL associated with the use of combination ipilimumab-nivolumab therapy. Therefore, in this paper, we describe a case of AGL associated with combination immunotherapy, highlighting its clinical course and key features in management.

## Case Presentation

This is the case of a 48-year-old female, with a complex oncological history of recurrent metastatic melanoma dating from 2004. She initially presented with choroidal melanoma with metastases to the retro-orbital soft tissue and right lung, and she underwent a left orbital exenteration and lung wedge resection. In 2007-2008, there was evidence of recurrence to the left orbit and the right lower lobe of the lung, leading to tumor resection and adjuvant local radiation. Remission was achieved from 2008 until 2019, when positron emission tomography–computed tomography (PET-CT) imaging showed a recurrence of lung metastasis. Therefore, nivolumab immunotherapy was initiated, where she underwent 23 cycles (May 2019 to February 2021), after which her disease was in temporary remission. However, a recurrence of metastatic disease was detected in the right lung in 2022 and in a left peri-renal mass in 2023. Nivolumab-ipilimumab combination immunotherapy was initiated in April 2023 and discontinued after 11 cycles (February 2024) in light of a complete metabolic response on PET imaging and due to the complications described below.

Other significant past medical history includes primary hypothyroidism diagnosed in 2002 with levothyroxine treatment remaining relatively stable during ICI therapy.

The patient's principal complaints of muscle aches, fevers, chills, worsening fatigue, and progressive lower extremity edema began in September 2023. PET-CT whole-body surveillance imaging (performed every 3-6 months) in December 2023 reported new diffuse soft tissue edema with minimal fluorodeoxyglucose uptake. An echocardiogram showed normal ventricular function with no structural defects.

## Diagnostic Assessment

She was referred to rheumatology for consideration of a rheumatological irAE. Although antinuclear antibodies were mildly positive 1:160 (speckled pattern), her complement (C3 and C4) levels were normal while antibodies to double-stranded deoxyribonucleic acid (dsDNA) and Smith (Sm) were negative. Also, all other related antibodies to Sjögren syndrome, systemic sclerosis, and undifferentiated connected tissue disease were negative. Aldolase was mildly elevated at 10.5 Units/L (SI 175 nkat/L) (normal reference, <7.7 Units/L; SI <128.36 nkat/L). Based on the preceding results, a rheumatological etiology was unlikely.

She was also worked up for adrenal insufficiency, whereby 10 Am adrenocorticotrophic hormone (ACTH) was 56.2 pg/mL (SI 12.36 pmol/L) (normal reference, 7.2-63.3 pg/mL; SI 1.58-13.9 pmol/L), cortisol 13.5 µg/dL (SI 372.6 nmol/L) (normal reference, 7-9 Am 4.3-22.4 µg/dL; SI 118.68-618.24 nmol/L) and dehydroepiandrosterone sulfate (DHEA-S) 194 µg/dL (SI 5.24 µmol/L) (normal reference, 35-256 µg/dL; SI 0.95-6.9 µmol/L).

**Figure 1.**
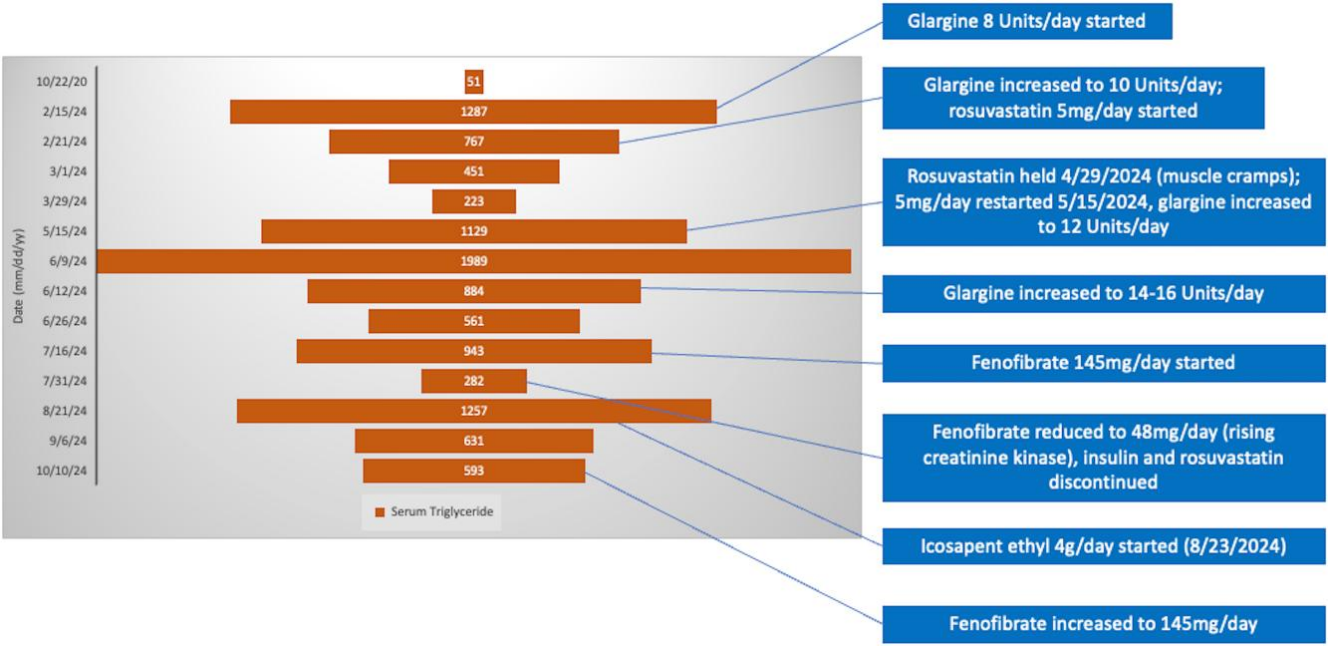
Trends of triglycerides (orange bars) during the course of insulin and lipid lowering therapy. (Dates in the left column are in the format month (mm)/day (dd)/year (yy); numbers are in mg/dL, conversion to SI units (mmol/L): mg/dL × 0.0113).

**Figure 2.**
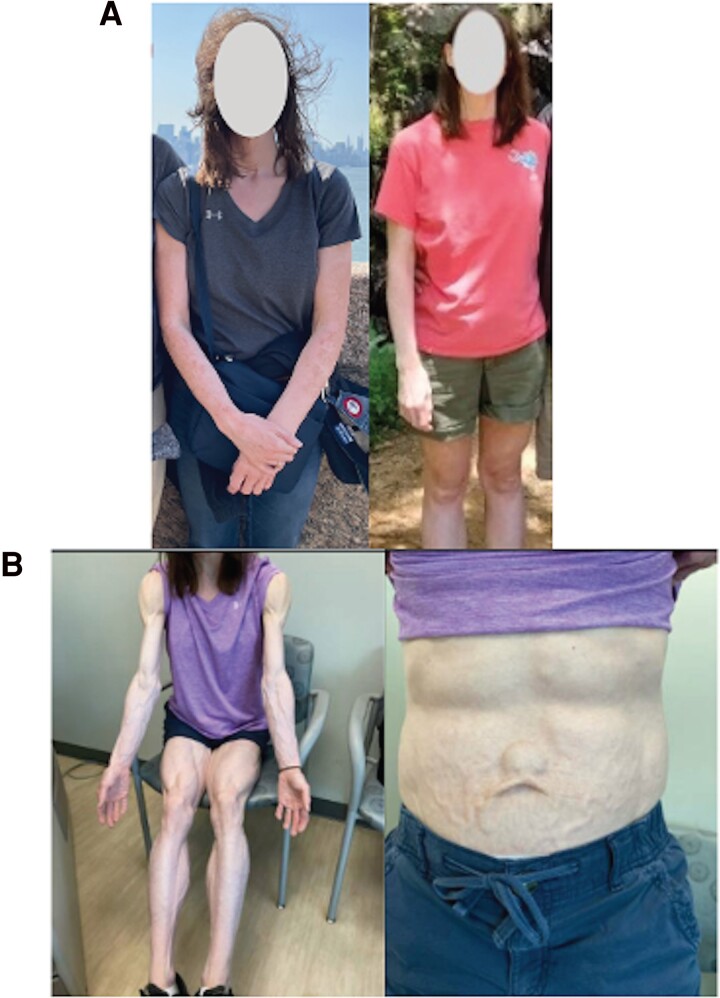
Patient prior to (A) and after (B) ipilimumab-nivolumab combination therapy, demonstrating significant generalized loss of adipose tissue, and resultant appearance of prominent veins and musculature.

In February 2024, the patient was found to have a hemoglobin A1c of 6.7% (reference, ≥ 6.5% diagnostic for diabetes mellitus) and a complete metabolic panel that was reported to be “lipemic.” A lipid panel subsequently drawn resulted a triglyceride level of 1287 mg/dL (SI 14.54 mmol/L) (normal reference, < 500 mg/dL: SI < 5.65 mmol/L). Also, high-density lipoprotein (HDL) had declined from 65 mg/dL (SI 1.683 mmol/L) (normal reference, >50 mg/dL; SI > 1.2 mmol/L) in 2020 to 26 mg/dL (SI 0.673 mmol/L). She was evaluated by endocrinology, where physical examination revealed significant loss of adiposity in the extremities. AGL was diagnosed based on physical examination findings, significant hypertriglyceridemia, and new onset diabetes mellitus. ICI therapy was discontinued shortly thereafter in March 2024. Rosuvastatin was commenced; however, triglycerides worsened (Fig. 1) with peak triglyceride level of 1989 mg/dL (SI 22.47 mmol/L) and lowest HDL of 18 mg/dL (SI 0.466 mmol/L). She was therefore referred to a tertiary Endo-oncology clinic for further consultation. There, physical examination revealed significant subcutaneous fat loss of the face, buttocks, and extremities with prominent veins and musculature (Fig. 2). Her body mass index (BMI) at the time was 21.17 kg/m^2^.

**Figure 3.**
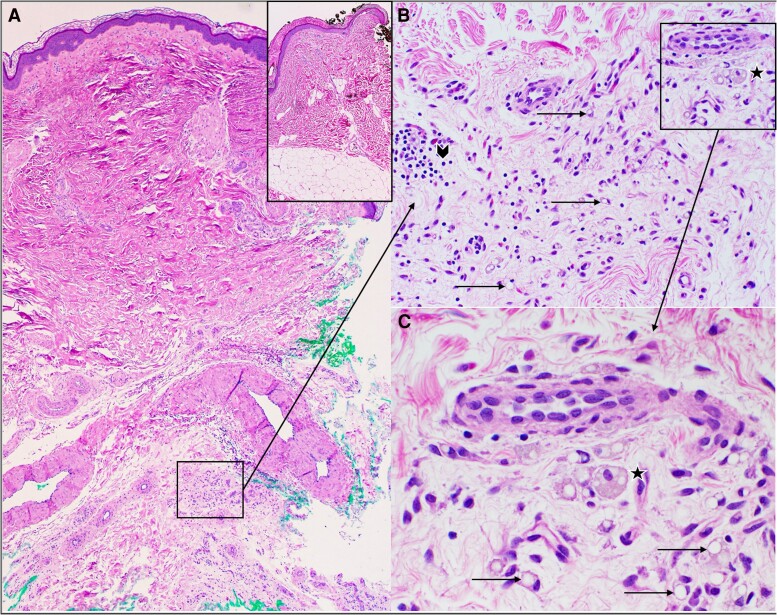
A, Low-power view (4×, hematoxylin and eosin [H&E]), of patient's excisional right leg skin biopsy, demonstrating epidermis, dermis, and atrophic subcutaneous fat. Inset of A (4×, H&E): Low-power view of age-, gender-, and site-matched skin excision demonstrating epidermis, dermis, and normal subcutaneous fat (normal control). B, Medium-power view (20×, H&E) of dermal-subcutaneous junction demonstrating involuting subcutaneous fat (arrows) in a background of lymphocytes (arrowhead) and lipophages (star). C, High-power view (40× H&E) of lipophage (star) and involuting subcutaneous fat (arrows).

Further work up included leptin and adiponectin levels which were < 0.5 ng/mL (SI < 0.5 µg/L) (normal reference, 2.8-15.6 ng/mL; SI 2.8-15.6 µg/L for BMI 21 kg/m^2^; patient BMI 20.65) and < 0.2 µg/mL (normal reference, 5-37 µg/mL for BMI < 25 kg/m^2^; patient BMI 20.65) respectively, and a lipoprotein(a) of 40.6 nmol/L (SI 0.0406 µmol/L) (normal reference, < 75 nmol/L; SI < 0.075 µmol/L). An excisional skin biopsy of the right leg was performed which showed changes consistent with involutional lipoatrophy/lipodystrophy (Fig. 3). PET-CT imaging performed for melanoma surveillance reported diffuse hepatic steatosis and paucity of mesenteric fat.

## Treatment

For the treatment of diabetes and hypertriglyceridemia, glargine insulin was commenced with subsequent addition of rosuvastatin 5 mg daily. Continuous glucose monitoring was done with average blood glucose 129 mg/dL (SI 7.2 mmol/L) (normal reference, 70-99 mg/dL; SI 3.9-5.5 mmol/L) during the first 2 to 4 weeks of insulin therapy. A significant reduction in triglycerides from 1287 mg/dL (SI 14.54 mmol/L) to 223 mg/dL (SI 2.52 mmol/L) was observed 6 weeks later (Fig. 2); however, symptoms of generalized myalgias occurred and rosuvastatin was briefly discontinued. With worsening triglycerides, rosuvastatin was re-introduced with addition of fenofibrate therapy. Creatine kinase was monitored, with maximal elevation to 340 Units/L (SI 5.578 µkat/L) (normal reference, 30-220 Units/L; SI 0.501-3.674 µkat/L). Insulin and rosuvastatin were ultimately discontinued, with dose of fenofibrate reduced to 48 mg/day and icosapent ethyl 2 g twice daily added.

## Outcome and Follow-Up

A most recent PET-CT whole-body scan showed a persistent focal uptake in the left ventricle concerning for cardiac metastasis, which was confirmed with cardiac magnetic resonance imaging. Current treatment plan is apical resection, with consideration of immunotherapy re-initiation depending on pathology and postoperative course.

## Discussion

Lipodystrophy syndromes are a rare heterogenous group that can be defined as a state of selective deficiency of adipose tissue in the absence of nutritional deprivation or catabolic state [1]. AGL, also known as *Lawrence syndrome*, is reported to be associated with viral infection and is strongly associated with autoimmunity [3-5]. In relation to immunotherapy, AGL is a rare irAE, with PD-1 monotherapy currently having the highest reported incidence [5-9]. Although it is a well-known phenomenon that combination immunotherapy increases the incidence and severity of endocrine irAEs [10], there are no current reports of AGL with combination immunotherapy. Our case therefore raises the possibility of combination immunotherapy-associated AGL, as the patient had no evidence of AGL with PD-1 monotherapy and only developed symptoms following the initiation of combination immunotherapy.

Firm diagnostic criteria of AGL is not established; however, it is generally accepted that key features of AGL include evidence of generalized loss of adipose tissue (either by physical examination or supported imaging such as dual energy x-ray absorptiometry or whole-body magnetic resonance imaging), insulin resistance, severe hypertriglyceridemia, and steatohepatitis in a nonobese individual [1, 5], which were all present in our patient. Panniculitis can occur in up to 25% of patients with AGL [11]. In retrospect, it is highly probable that our patient's generalized edema and subcutaneous swelling noted on PET-CT imaging were panniculitis associated with AGL. Interestingly, one other case report highlighted a markedly similar presentation with incidental subcutaneous edema picked up on surveillance PET-CT whole-body scan—suggestive of panniculitis [7]. This report further theorizes the possibility of generalized subcutaneous inflammation as an early feature of AGL, and the high index of suspicion warranted in the event such a finding is detected on surveillance imaging. Although leptin assessment is not routinely recommended for diagnosis, hypoleptinemia can support suspicion and guide medical management [1].

Leptin replacement therapy's role in the management of lipodystrophy has long been established, based on in vivo studies and small clinical trials [12]. This led to the introduction of metreleptin (recombinant human methionyl leptin), which is currently the only drug approved for lipodystrophy [13]. It is approved in the United States as a first line adjunct to diet for treatment of metabolic complications in patients with generalized lipodystrophy [1]. With its potential benefits, however, comes great concern for potential side effects, which can occur in approximately 30% of patients [1, 14, 15], of which hypoglycemia, weight loss, and hypersensitivity reactions are the more common adverse events. In addition, neutralizing antibodies may develop, leading to treatment failure and sepsis [16]. Finally, there is concern of cancer risk with metreleptin therapy, particularly T-cell lymphoma, although questions arise as to whether this correlation is related to metreleptin or generalized lipodystrophy syndromes in general [1, 15]. Given the severe side effects associated with metreleptin therapy, patients considered for treatment must be enrolled through a Risk Evaluation and Mitigation Strategy Program. Therefore, for our patient, due to its potential side effect burden in comparison to insulin combined with lipid lowering agents alone, it was decided to forego metreleptin therapy.

## Learning Points

- Panniculitis may be an early feature of ICI-associated AGL which may be incidentally reported on PET-CT whole-body scan imaging.
- Hypoleptinemia may support the diagnosis of AGL.
- Although there may be a strong association with PD-1 inhibitors and ICI-induced AGL, it is possible that combination immunotherapy increases the risk of AGL, as with other ICI related endocrinopathies.
- Although metreleptin is the only FDA-approved drug for AGL, its broad side effect profile should be considered while determining its utility in ICI related lipodystrophy.
- This case further emphasizes the need for a team-based approach in cancer therapy, which includes relevant screening for adverse events as well as early referral to adjunct specialty care.

## Contributors

All authors made individual contributions to authorship. A.S. and A.K.S.S. were involved in the diagnosis and ongoing management of the patient. R.K., C.L.M., M.V.D., A.K.S.S., and A.S. were involved in data collection and preparation of the final draft. M.L. was involved in the preparation of histopathology images. All authors critically reviewed the manuscript and approved the final draft.

## Data Availability

Some or all data sets generated during and/or analyzed during the present study are not publicly available but are available from the corresponding author on reasonable request.

## Abbreviations

AGL: acquired generalized lipodystrophy
BMI: body mass index
HDL: high-density lipoprotein
ICI: immune checkpoint inhibitor
irAE: immune-related adverse event
PD-1: programmed cell death protein 1
PET-CT: positron emission tomography–computed tomography
